# Challenges, successes and patterns of enrolment in the INSIGHT Strategic Timing of AntiRetroviral Treatment (START) trial

**DOI:** 10.1111/hiv.12229

**Published:** 2015-02-25

**Authors:** J Grarup, C Rappoport, NW Engen, C Carey, F Hudson, E Denning, S Sharma, E Florence, MJ Vjecha, International Network for Strategic Initiatives in Global HIV Trials (INSIGHT) START Study Group

**Affiliations:** ^1^ Copenhagen HIV Programme, RigshospitaletUniversity of CopenhagenCopenhagenDenmark; ^2^ University of California, San Francisco DOM/HIV/AIDS DivisionSan Francisco General HospitalSan FranciscoCAUSA; ^3^ Coordinating Centers for Biometric ResearchDivision of BiostatisticsSchool of Public HealthUniversity of MinnesotaMinneapolisMNUSA; ^4^ Kirby InstituteUniversity of New South WalesSydneyAustralia; ^5^ Medical Research Council Clinical Trials Unit at UCLUniversity College LondonLondonUK; ^6^ Institute of Tropical MedicineAntwerpBelgium; ^7^ Institute for Clinical Research, Inc.Veterans Affairs Medical CenterWashingtonDCUSA

**Keywords:** antiretroviral therapy initiation, enrolment, HIV/AIDS, START trial

## Abstract

**Objectives:**

The aim of this report is to describe the challenges, successes and patterns of enrolment in the Strategic Timing of AntiRetroviral Treatment (START) study.

**Methods:**

START is a collaboration of many partners with central coordination provided by the protocol team, the statistical and data management centre (SDMC), the International Network for Strategic Initiatives in Global HIV Trials (INSIGHT) network leadership, international coordinating centres and site coordinating centres. The SDMC prepared reports on study accrual, baseline characteristics and site performance that allowed monitoring of enrolment and data quality and helped to ensure the successful enrolment of this large international trial. We describe the pattern of enrolment and challenges faced during the enrolment period of the trial.

**Results:**

An initial pilot phase began in April 2009 and established feasibility of accrual at 101 sites. In August 2010, funding approval for an expanded definitive phase led to the successful accrual of 4688 participants from 215 sites in 35 countries by December 2013. Challenges to accrual included regulatory delays (e.g. national/local ethics approval and drug importation approval) and logistical obstacles (e.g. execution of contracts with pharmaceutical companies, setting up of a central drug repository and translation of participant materials). The personal engagement of investigators, strong central study coordination, and frequent and transparent communication with site investigators, community members and participants were key contributing factors to this success.

**Conclusions:**

Accrual into START was completed in a timely fashion despite multiple challenges. This success was attributable to the efforts of site investigators committed to maintaining study equipoise, transparent and responsive study coordination, and community involvement in problem‐solving.

## Introduction

The Strategic Timing of AntiRetroviral Treatment (START) study is a pivotal, large, international randomized clinical trial designed to assess the optimal timing for the initiation of antiretroviral treatment (ART) in ART‐naïve HIV‐positive individuals. The study overcame multiple challenges in the accrual of 4688 ART‐naïve study participants willing to be followed for at least 3 years to ascertain key clinical endpoint events. We describe the patterns of enrolment observed, the challenges encountered and resolved, and the factors that contributed to the successful and timely accrual of this global trial.

Details of the rationale and design of the START study have been published previously 1. The history of the trial and community member involvement in its design and implementation are summarized elsewhere in this supplement 2, 3.

## Methods

The sources used to describe the successful enrolment of the START study are: (1) the experienced staff at the four international coordinating centres (ICCs) and the statistical and data management centre (SDMC) of the International Network for Strategic Initiatives in Global HIV Trials (INSIGHT), based at the University of Minnesota, Minneapolis, MN, USA; and (2) centrally collected data on site establishment and study accrual.

**Figure 1:**
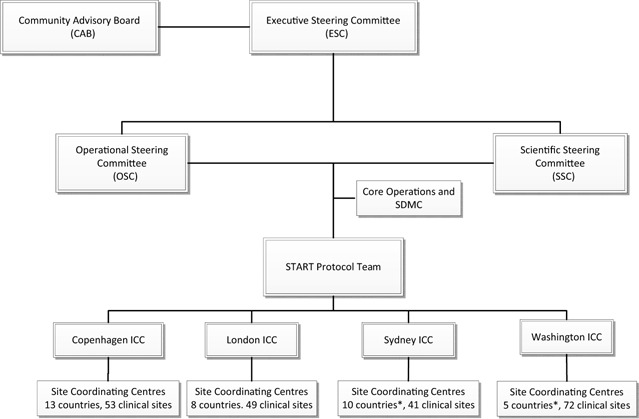
Operational structure of the International Network for Strategic Initiatives in Global HIV Trials (INSIGHT) network and Strategic Timing of AntiRetroviral Treatment (START) study governance. SDMC, statistical and data management centre; ICC, international coordinating centre. *Both the Sydney ICC and the Washington ICC have sites in South Africa.

The START study is a collaboration of many organizations, funders and pharmaceutical donors. Central coordination is provided by the protocol team, the SDMC, INSIGHT network leadership, and four experienced ICCs with investigators and staff at site coordinating centres (SCCs) and sites in 35 countries (Fig. 1). A detailed network website provided publicly accessible up‐to‐date reports on main study and substudy accrual, baseline data, and site performance, in addition to password‐protected access to study case report forms and data queries, which allowed partners to monitor and maintain quality to ensure the successful enrolment of this large complex trial.

## Results

### Enrolment patterns

#### Two phases of enrolment

**Figure 2:**
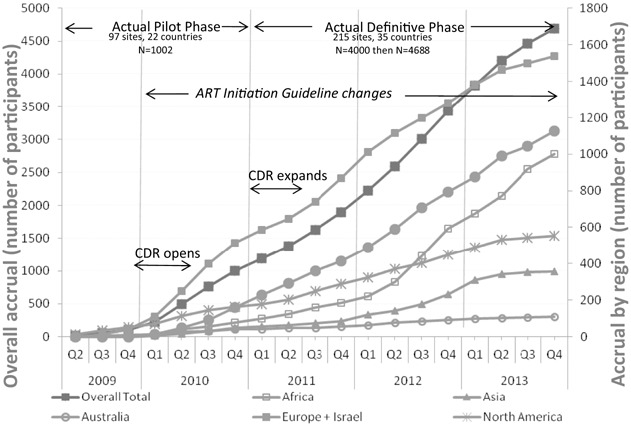
Cumulative quarterly enrolment in the Strategic Timing of AntiRetroviral Treatment (START) trial overall and by region. ART, antiretroviral therapy; CDR, Central Drug Repository; Q, quarter of the year.

Accrual into the START study proceeded in two phases. The National Institute of Allergy and Infectious Diseases (NIAID), the primary funder, required an initial pilot phase to establish feasibility of accrual with a goal of 900 participants. This phase commenced after the first participant was enrolled in April 2009 and, overall, included 101 sites in 22 countries. Of these sites, 97 had enrolled 1002 participants by the end of 2010. In August 2010, with steady accrual at an average of 87 participants per month, NIAID approved the expansion of the study into the definitive phase with an initial goal of 4000 participants. A protocol‐specified sample‐size re‐estimation at the end of 2012 increased the initial enrolment goal to 4600. By December 2013, accrual into START was successfully completed with 4688 participants enrolled at 215 sites in 35 countries. Cumulative enrolment over time is presented in Figure 2.

The purpose of the pilot phase was to determine whether sites could sufficiently engage and obtain consent from volunteers – all reasonably healthy and most relatively new to HIV care and acceptance of an HIV diagnosis – to be randomized either to start ART immediately or to defer ART according to the protocol. The pilot phase demonstrated that enrolling over 900 participants in a limited number of sites was feasible within 1 year, once study drugs became available to sites from the Central Drug Repository (CDR).

The diversity of sites selected for the pilot phase was limited, although all had successfully enrolled participants in previous long‐term trials and claimed to have access to sufficient numbers of ART‐naïve patients with high CD4 cell counts. Most pilot‐phase sites were urban. Many of these sites were at specialized clinics where potential participants were mostly men who had sex with men and had access to ART through private insurance or government programmes. The expansion of the trial with 114 new sites enrolling in the definitive phase increased the diversity of sites and countries, resulting in a higher percentage of women and people of colour enrolled as study participants.

The sample‐size re‐estimation reduced the target number of primary event endpoints from 370 to 213. The minimum average follow‐up of 3 years after completion of accrual remained unchanged. The primary reason for the changes in event target and sample size were: (1) the median baseline CD4 cell count was higher than originally planned; and (2) the pooled primary event rate was lower than assumed. The cochairs also decided to modify the inclusion criterion for age once accrual reached 4000 to enrol only persons aged 35 years or older, a population with a higher risk of primary events. These changes were approved by NIAID and presented to the study's independent Data and Safety Monitoring Board (DSMB). The new age criterion took effect on 16 July 2013.

#### Enrolment by region and country

Fourteen countries each enrolled over 100 participants and account for 85% of all participants, as shown in Table 1. Overall, accrual was geographically diverse with enrolment on six continents. Europe was the top enrolling region with one‐third of all study participants. Sites in Europe, South America/Mexico, and Africa accrued 79% of the study population. The highest enrolling sites were in South America (Argentina, Brazil, Chile and Peru), Africa (South Africa and Uganda), and Asia (Thailand), as shown in Table 2.

**Table 1:** Enrolment in the Strategic Timing of AntiRetroviral Treatment (START) trial by region and country

Europe and Israel 1539 participants (32.8%*) 101 sites, 20 countries		South America and Mexico 1174 participants (25.0%*) 24 sites, 5 countries		Africa 1002 participants (21.4%*) 10 sites, 5 countries		North America 507 participants (10.8%*) 55 sites, 1 country		Asia and Australia 466 participants (9.9%*) 25 sites, 4 countries	
Country (*n* = sites)	*N*	Country (*n* = sites)	*N*	Country (*n* = sites)	*N*	Country (*n* = sites)	*N*	Country (*n* = sites)	*N*
UK (21)	339	Brazil (7)	619	South Africa (5)	518	USA (55)	507	Thailand (9)	248
Germany (17)	312	Argentina (10)	216	Uganda (2)	349			Australia (13)	109
Spain (12)	234	Peru (5)	215	Nigeria (1)	50			India (2)	91
France (12)	111	Chile (1)	76	Morocco (1)	44			Malaysia (1)	18
Belgium (4)	102	Mexico (1)	48	Mali (1)	41				
Greece (6)	101								
Poland (3)	68								
Portugal (4)	67								
Denmark (3)	33								
Italy (2)	33								
Switzerland (4)	31								
Israel (2)	28								
Finland (1)	23								
Norway (1)	15								
Czech Republic (2)	13								
Estonia (1)	8								
Austria (2)	7								
Ireland (1)	7								
Luxembourg (1)	5								
Sweden (2)	2								

*Percentage of total enrolment.

**Table 2:** Regional distribution of sites by enrolment stratum

Region	No. of sites	No. of participants				Median no. of participants enrolled by site (25%, 75%)
		< 10	10–20	21–50	> 50	
North America	55	35	15	5	0	8 (3, 14)
Europe and Israel	101	40	37	23	1	12 (6, 19)
South America and Mexico	24	4	4	8	8	35 (17, 70)
Australia	13	10	2	1	0	6 (5, 8)
Asia	12	1	6	3	2	18 (13, 31)
Africa	10	0	1	4	5	70 (41, 159)
Total	215	90	65	44	16	12 (6, 23)

In January 2011, the study cochairs decided to cap enrolment at 50 participants for sites in four countries endemic for tuberculosis (TB): India, Nigeria, Uganda and South Africa, out of concern that TB events in deferred‐arm participants might be numerous at these sites. In November 2012, at the time of the sample‐size re‐estimation, the cochairs lifted this cap.

In contrast to these high‐enrolling sites, 90 sites – mostly in the USA, Europe and Australia – enrolled only 437 participants, an average of less than five each; moreover, five countries enrolled less than 10 participants each (Table 1). Some of the low‐enrolling sites in the USA and Europe participated with their own funding, and some had limited accrual goals. Some sites that had enrolled vigorously in previous trials of treatment‐experienced participants found it difficult to accrue participants into START, particularly in the USA.

#### Impact of availability of study drug

Pilot‐phase site accrual began in 2009 at 30 sites that could access ART through local sources. Accrual accelerated once the CDR opened to some countries in late 2009 and additional countries in early 2010 and again once access to the CDR expanded in early 2011 for the definitive‐phase sites. As seen in Figure 2, the rate of enrolment was consistently dependent on access to the CDR.

Accrual continued to accelerate in the first half of 2012 and remained steady to June 2013. This was attributable mainly to the opening of high‐enrolling sites in Brazil, Africa and Asia that had been delayed because of regulatory and drug availability obstacles described below.

#### Impact of age cap

In the enrolment period after introduction of the age cap in July 2013, a total of 422 participants age ≥ 35 years were accrued, which is a decrease from 828 participants accrued in the previous half‐year period. Sites in Africa, South America/Mexico and Europe accrued the highest numbers of these older participants.

#### Accuracy of site enrolment projections

One criterion for site selection in START was a site's commitment to enrol a minimum of 25 participants by the end of the definitive phase. Some sites had projected a higher target. Sites in the USA and Europe consistently overestimated their ability to enrol the trial, while many sites in Africa, South America/Mexico and Asia exceeded their initial targets.

#### Impact of screening procedures

The original inclusion criterion requiring two consecutive CD4 cell counts > 500 cells/μL at least 2 weeks apart within 60 days before randomization resulted in a large number of screening failures and was amended early on to eliminate the requirement that the counts be consecutive. A total of 5816 individuals consented to randomization, but 1128 (19%) were not enrolled. Of these, 1013 were not eligible, and 115 were eligible but not randomized. Some heterogeneity was observed, with high proportions of consenting participants randomized in Asia, Australia and Europe; however, similar proportions were seen among gender, age and educational groups (Table 3). A complete assessment of the larger pool of individuals considered for START is not possible as no data were collected on the number of prescreened individuals prior to signing consent.

**Table 3:** Characteristics of participants: consenting to participate versus randomized

	Consenting	Randomized		Not randomized	
	*N*	*N*	%	*N*	%
Region					
Africa	1276	1002	79	274	21
Asia	392	357	91	35	9
Australia	124	109	88	15	12
Europe and Israel	1777	1539	87	238	13
North America	682	507	74	175	26
South America and Mexico	1565	1174	75	391	25
Age group					
< 35 years	2672	2121	79	551	21
≥ 35 years	3144	2567	82	577	18
Gender					
Male	4242	3429	81	813	19
Female	1574	1259	80	315	20
Education					
Less than high school/less than year 12/less than ‘A’ equivalent	1790	1399	78	391	22
High school graduate or equivalent/year 12/‘A’ level equivalent	1286	1018	79	268	21
Completed vocational training	499	417	84	82	16
Some college/some university	993	799	80	194	20
Received bachelor's degree/university degree/TAFE degree	951	806	85	145	15
Any postgraduate education	279	249	89	30	11
Unknown	18	0	0	18	100
**Total**	**5816**	**4688**	**81**	**1128**	**19**

TAFE, Technical and Further Education, New South Wales Institutes.

### Challenges

#### Critical role of the CDR and country depots

The setting up of the CDR was a complex and lengthy task. Two primary depots, operated by a private subcontractor, provided drugs directly to sites in the USA, Europe and several other countries. Continuity of drug supply was critical to the successful enrolment of the study. This involved drafting and printing labels for over 20 drug formulations in 19 separate languages and up to 42 local language versions, with annual review and revision for changes and newly added drugs. Each new drug and new drug formulation required the creation of new labels as well as changes to the web drug‐ordering system. Projection of drug usage, revision of clinical trial agreements (CTAs) with pharmaceutical companies, and timely arrival of the annual deliveries of donated drugs with varying expiry dates were major challenges. Because of the complexity of import requirements, seven country‐specific CDR depots were eventually established. To date, no major disruption in study drug supply has occurred.

#### Legal, administrative and regulatory challenges

The process of resolving issues related to indemnification, study sponsorship, and requirements for pharmacovigilance reporting to the European Union delayed implementation of the pilot phase for nearly a year, as resolution was a prerequisite for negotiating CTAs with the six pharmaceutical companies donating drugs for the CDR as well as execution of agreements with outside contractors and within the network 4.

The central and national translation of protocols, informed consent forms and other participant materials into up to 42 language variants demanded considerable time and expense. A number of countries utilized a central body for ethics review of study documents, which at times streamlined the review process, although this was offset by the need in other countries for additional review by a local committee. These regulatory processes took from 3 to 18 months.

The ICCs experienced growing administrative bureaucracy within hospitals, which was an increasingly important factor delaying opening of the study at some sites as a consequence of more negotiation rounds to settle contracts. Newly established legal departments have begun to require execution of agreements mainly to protect hospital interests in industry‐sponsored clinical trials. These departments, however, do not always distinguish between investigator‐driven academic research and industry‐sponsored trials, resulting in unrealistic requirements and fees that a publicly funded research network cannot accommodate.

Differing health systems and structures across countries also affected recruitment. In some countries, health care services were primarily provided by private general practitioners, which hampered recruitment at sites relying on referrals from private practices that in the current environment preferred to hold on to their patients.

The financial crisis that hit Europe also affected enrolment. Increased time was needed to obtain approval in some countries where ICCs reported that national regulatory and ethical boards were shut down for varying periods and site coordinating staff at hospitals were influenced by the government‐induced budget cuts, especially in Spain and Greece.

Competing studies involving treatment‐naïve participants also created a challenge for some sites, especially in Germany where study participants are prohibited by law from participating in more than one clinical study at a time, as they had to choose between START, a relatively complex study, and other studies, often shorter in length and perhaps better funded.

In the definitive phase, START added 114 sites enrolling in 35 countries – of which 13 sites were new to the INSIGHT network – which required an increased mobilization of ICC, SCC and network resources to deal with the lengthy processes for regulatory approval and drug importation and delayed the onset of accrual at many sites with the potential for high enrolment. While ICCs and SCCs tried not to delay a site's opening to the main study because of regulatory issues with a substudy, competition for ICC and SCC resources in setting up the higher intensity substudies may have delayed opening to the main study at some sites.

**Figure 3:**
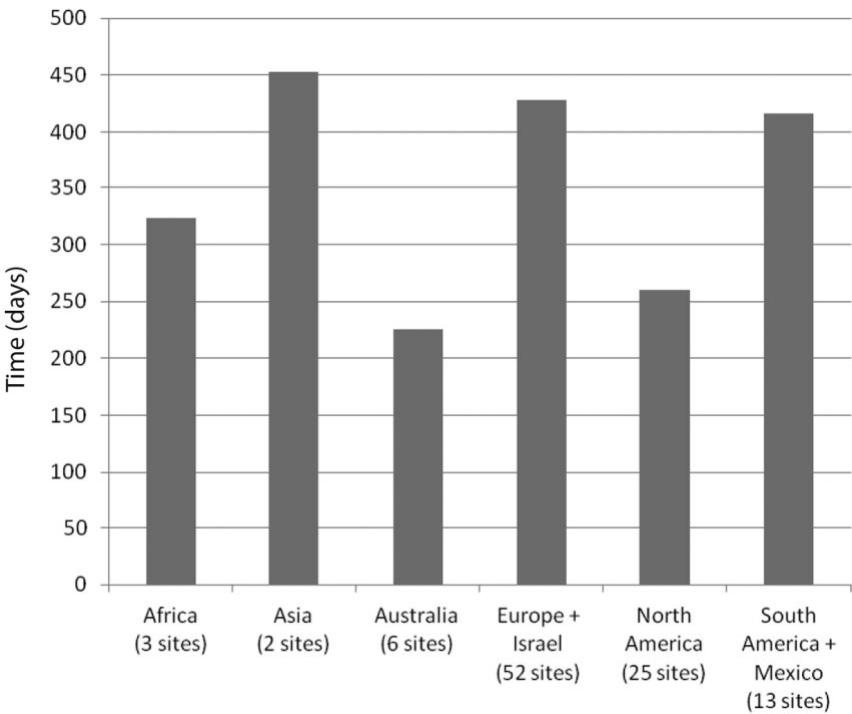
Time from finalization of the study protocol to pilot sites opening, after regulatory and network approval.

**Figure 4:**
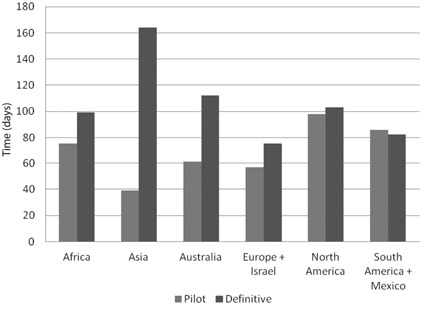
Days from site opening to first participant enrolled, after regulatory and network approval.

As seen in Figures 3 and 4, the aforementioned challenges impacted the time from finalization of the protocol to the first enrolment by a site. The median days to site opening in the pilot phase differed by region. Sites in the USA and Australia opened soonest. Although these sites were able to access antiretroviral drugs through local sources, in the end, accrual depended on availability of drugs from the CDR. Sites added in the definitive phase in countries that did not participate in the pilot phase, such as India, Malaysia, Nigeria and Uganda, had a longer time from site opening to first enrolment as a consequence of approvals needed to access CDR drugs, translate study documents and complete other country‐specific regulatory requirements.

#### Changes in HIV treatment guidelines and use of ART as prevention

The changes in HIV treatment guidelines on the timing of initiation of ART by several countries from 2010 to 2012 and by the World Health Organization in 2013, as well as the publication of results of HIV Prevention Trials Network (HPTN) 052 in 2011 5, posed challenges to START recruitment. Multiple letters to investigators were sent by study cochairs highlighting the lack of evidence from randomized controlled trials documenting the risks and benefits of early initiation of ART 3, 6. Equipoise regarding the START strategy remained intact among most site investigators, although there were a few notable exceptions at some sites where accrual dropped off.

### Factors contributing to success

The INSIGHT network included community representation at all stages of the development of the START study, from initial concept to completion of accrual 3. The INSIGHT Community Advisory Board (CAB) took the lead in creating easy‐to‐understand participant materials and fact sheets for both the main trial and its substudies. The CAB also wrote newsletters that were available to participants in each ICC after ethics approval. CAB members also wrote articles explaining the rationale for the study for use in their own countries 3, 7, 8.

Understanding that many prospective study participants may have recently been diagnosed with HIV, INSIGHT CAB members worked with network leadership and the ICCs in developing nontraditional ways for sites to reach out to potential participants. The Washington ICC produced a website (http://www.thestartstudy.org/) and recruitment video in close collaboration with the local CAB and other community members to recruit participants in the USA.

Establishment of efficient operational structures as depicted in Figure 1, as well as engagement in frequent and transparent communication at all levels of the study, were key to the success of START accrual. Frequent and inclusive team phone conferences were critically important for problem‐solving and consensus decision‐making, including input from the CAB. The coordinated efforts by the protocol team, network leadership, and ICC staff in conjunction with site investigators, site research staff, and community member advocates all contributed to the success in addressing multiple challenges to study accrual over time.

### Substudies

To avoid a negative impact on the main trial, the START protocol team developed guidance in 2009 to assist sites in making decisions about balancing the desire to participate in substudies 9, 10, 11, 12, 13, 14 with the necessity of maintaining enrolment and data quality in the main study. Table 4 shows the number of sites that participated in the less intensive Informed Consent and Genomics substudies and the number that conducted one or more of the moderate‐ to high‐intensity substudies: Neurology, Arterial Elasticity, Pulmonary, and Bone Mineral Density. In addition, the table shows average enrolment in the main study at sites participating in one or more substudies. Of note, some participants were enrolled in the main study prior to the opening of substudy enrolment.

**Table 4:** Site participation in Strategic Timing of AntiRetroviral Treatment (START) substudies and average enrolment in the main study

Substudies	No. of sites participating	% of all sites (*n* = 215)	Average no. of participants enrolled in main study
Genomics	155	72	18
Informed Consent	151	70	24
1 moderate‐high intensity*	87	40	25
2 moderate‐high intensity*	32	15	38
≥ 3 moderate‐high intensity*	8	4	32

*Moderate to high intensity substudies were Neurology, Arterial Elasticity, Pulmonary, and Bone Mineral Density.

## Discussion

Enrolment took place largely within the 1‐year pilot phase after drugs became available from the CDR and the 3‐year definitive phase prior to the sample‐size re‐estimation. As seen in Table 1 and Figure 2, high enrolment was achieved at sites in South America, Africa and Asia. This was the case despite the fact that these sites started enrolling relatively late and encountered many obstacles that delayed enrolment, for example, drug importation challenges and the protocol team‐imposed limitation of enrolment to 50 participants per site in four TB‐endemic countries.

Possible reasons for the difference in accuracy of enrolment projections by region may be the effect of changes in HIV treatment guidelines, lack of equipoise among investigators and potential participants in different countries regarding randomization, and lower numbers of treatment‐naïve participants at sites in the USA and Europe. The relatively many sites that enrolled fewer than 10 participants demonstrate how difficult it is to predict which sites will accrue well. The process of setting up one or more sites in a new country, however, is complex, time‐consuming and costly, all of which must be weighed against potential accrual and contribution to a trial's geographical diversity.

Ensuring donation of adequate supplies of antiretroviral drugs with long shelf life from pharmaceutical companies was a key critical factor for the successful completion of enrolment.

Initiation of substudies competing for site resources was a concern for the ICCs. However, sites seemed to be realistic about their ability to manage substudies effectively, as sites that signed up for three or more intensive substudies did accrue on average as many participants in START as sites only involved in a single substudy.

Multiple approaches were applied to assist sites in resource‐rich settings to inform and recruit treatment‐naïve, HIV‐positive persons. General community and site CAB support and the many initiatives to develop brochures, web‐based video and other participant materials, as well as organizing local events to train investigators and enlarge the pool of possible study referrals, were crucial.

Coordinating centres observed that enrolment was greater when the site leader, coinvestigators, and key research staff remained convinced about the continued relevance of the questions being asked. The timing of study opening was important. Some sites reported that potential participants they had lined up and included in their initial recruitment estimates were lost during the wait for the CDR to open.

Some voiced concern that START could not be enrolled in the face of changing HIV treatment guidelines. That START continued to accrue, despite pressure on some investigators and deferred‐arm participants to initiate ART earlier than specified in the protocol, is an important achievement. While HPTN 052 and other trials provided welcome evidence for the use of ART to prevent transmission 15, START will confirm the risks and benefits of earlier ART use for the individual. Such evidence is urgently needed to inform future global and national health policies to end the HIV epidemic.

For years, the community has demanded that a randomized controlled trial to answer the ‘when to start’ question be funded. Now that the study is fully accrued, all involved will continue to work to ensure the successful completion of this pivotal HIV treatment strategy trial.

The START study is registered at clinicaltrials.gov (NCT00867048).

## Funding

The START study is primarily funded by the National Institute of Allergy and Infectious Diseases of the National Institutes of Health under Award Number UM1‐AI068641, the Department of Bioethics at the NIH Clinical Center and five NIH institutes: the National Cancer Institute, the National Heart, Lung, and Blood Institute, the National Institute of Mental Health, the National Institute of Neurological Disorders and Stroke and the National Institute of Arthritis and Musculoskeletal Disorders. Financial support is also provided by the French Agence Nationale de Recherches sur le SIDA et les Hépatites Virales (ANRS), the German Ministry of Education and Research, the European AIDS Treatment Network (NEAT), the Australian National Health and Medical Research Council, and the UK Medical Research Council and National Institute for Heath Research. Six pharmaceutical companies (AbbVie, Inc., Bristol‐Myers Squibb, Gilead Sciences, GlaxoSmithKline/ViiV Healthcare, Janssen Scientific Affairs, LLC, and Merck Sharp and Dohme Corp.) donate antiretroviral drugs to START.

## Disclosures

The content is solely the responsibility of the authors and does not necessarily represent the official views of the National Institutes of Health. The University of Minnesota, the sponsor of START, receives royalties from the use of abacavir, one of the HIV medicines that can be used in START. Potential conflicts of interest: EF reports unrestricted research grants from ViiV Healthcare, MSD, Gilead, J&J, and BMS and travel grants from Gilead. The other authors report no conflicts of interest.
